# Correlation between noncommunicable disease mortality in people aged 30–69 years and those aged 70–89 years

**DOI:** 10.2471/BLT.18.227132

**Published:** 2019-06-24

**Authors:** Peter Byass

**Affiliations:** a Department of Epidemiology and Global Health, Umeå University, 90187 Umeå, Sweden.

## Abstract

**Objective:**

To investigate whether the key metric for monitoring progress towards sustainable development goal target 3.4 that is measuring premature noncommunicable disease mortality (deaths among people aged 30–69 years), is ageist.

**Methods:**

To examine the relationship between premature noncommunicable disease mortality and noncommunicable disease mortality in older people, a database of mortality rates for cardiovascular disease, cancer, chronic obstructive pulmonary disease and diabetes in people aged 30 to 69 years and 70 to 89 years was compiled using estimates from the Global Burden of Disease Study 2017. The data covered 195 countries, six time-points and both sexes, giving 2340 instances. The World Health Organization’s (WHO’s) life-table method for the premature noncommunicable disease mortality metric was applied to the data.

**Findings:**

There was a strong correlation between noncommunicable disease mortality patterns in the premature and older age groups, which suggests that measuring premature noncommunicable disease mortality is informative about such mortality in later life. Neither time nor geographical location had a substantial effect on this correlation. However, there were female-to-male differences in age-specific probabilities of death due to noncommunicable disease, implying that noncommunicable disease mortality should be assessed using a sex-disaggregated approach.

**Conclusion:**

As the established WHO metric for premature noncommunicable disease mortality was predictive of noncommunicable disease mortality in older people, the metric should not be construed as ageist. Focusing resources on measuring premature noncommunicable disease mortality will be appropriate, particularly in settings without universal civil death registration. This approach should not prejudice the provision of health services throughout the life-course.

## Résumé

**Objectif:**

Rechercher si l'indicateur clé permettant de suivre la progression par rapport à la cible 3.4 des Objectifs de développement durable, qui mesure la mortalité prématurée due à des maladies non transmissibles (décès parmi les personnes de 30 à 69 ans), fait preuve d'âgisme.

**Méthodes:**

Afin d'examiner la relation entre la mortalité prématurée due à des maladies non transmissibles et la mortalité de personnes plus âgées due à ce même type de maladies, nous avons établi une base de données répertoriant les taux de mortalité due à des maladies cardiovasculaires, au cancer, à la bronchopneumopathie chronique obstructive et au diabète chez les personnes âgées de 30 à 69 ans et de 70 à 89 ans, à l'aide d'estimations de l'étude 2017 sur la charge mondiale de morbidité (Global Burden of Disease Study). Les données portaient sur 195 pays, six points temporels ainsi que sur les deux sexes, et donnaient 2340 exemples. La méthode des tables de mortalité de l'Organisation mondiale de la Santé (OMS), employée pour l'indicateur de la mortalité prématurée due à des maladies non transmissibles, a été appliquée à ces données.

**Résultats:**

Il est apparu une forte corrélation entre les schémas de mortalité due à des maladies non transmissibles des deux groupes d'âge, ce qui suggère que la mesure de la mortalité prématurée due à des maladies non transmissibles renseigne sur la mortalité pour les mêmes causes à un âge plus avancé. Ni le temps ni le lieu géographique n'avaient d'effet notable sur cette corrélation. En revanche, on a observé des différences entre hommes et femmes dans les probabilités de décès dû à des maladies non transmissibles selon l'âge, ce qui implique qu'il faudrait évaluer la mortalité due à des maladies non transmissibles en différenciant les sexes.

**Conclusion:**

Étant donné que l'indicateur établi par l'OMS pour la mortalité prématurée due à des maladies non transmissibles permettait de prédire la mortalité des personnes plus âgées due à ce même type de maladies, il conviendrait de ne pas interpréter cet indicateur comme faisant preuve d'âgisme. Il sera judicieux de concentrer les ressources sur la mesure de la mortalité prématurée due à des maladies non transmissibles, en particulier dans les régions qui n'ont pas de système universel d'enregistrement des décès. Cette démarche ne doit pas nuire à la fourniture de services de soins tout au long de la vie.

## Resumen

**Objetivo:**

Investigar si el indicador clave para monitorear el progreso hacia la meta 3.4 de desarrollo sostenible, que es la medición de la mortalidad prematura por enfermedades no transmisibles (muertes entre personas de 30 a 69 años de edad), es el de la edad.

**Métodos:**

Para examinar la relación entre la mortalidad prematura por enfermedades no transmisibles y la mortalidad por enfermedades no transmisibles en las personas mayores, se compiló una base de datos de las tasas de mortalidad por enfermedades cardiovasculares, cáncer, enfermedades pulmonares obstructivas crónicas y diabetes en personas de 30 a 69 años y de 70 a 89 años de edad, utilizando las estimaciones del Estudio sobre la Carga Mundial de las Enfermedades 2017. Los datos abarcaban 195 países, seis puntos temporales y ambos sexos, lo que representaba 2 340 casos. Se aplicó a los datos el método de la tabla de mortalidad prematura por enfermedades no transmisibles de la Organización Mundial de la Salud (OMS).

**Resultados:**

Hubo una fuerte correlación entre las pautas de mortalidad por enfermedades no transmisibles en los grupos de edad prematura y de edad avanzada, lo que indica que la medición de la mortalidad prematura por enfermedades no transmisibles es informativa sobre esa mortalidad en etapas posteriores de la vida. Ni el tiempo ni la ubicación geográfica tuvieron un efecto sustancial en esta correlación. Sin embargo, existen diferencias entre las mujeres y los hombres en cuanto a las probabilidades específicas de muerte por enfermedades no transmisibles, lo que implica que la mortalidad por enfermedades no transmisibles debe evaluarse mediante un enfoque desagregado por sexo.

**Conclusión:**

Dado que el indicador establecido por la OMS para la mortalidad prematura por enfermedades no transmisibles es predictivo de la mortalidad por enfermedades no transmisibles en las personas de edad, el indicador no debe interpretarse como un indicador de la edad. Será apropiado centrar los recursos en la medición de la mortalidad prematura por enfermedades no transmisibles, en particular en los entornos en los que no existe un registro universal de defunciones civiles. Este enfoque no debe perjudicar la prestación de servicios sanitarios a lo largo de toda la vida.

## ملخص

**الغرض:**

التحقق مما إذا كان المقياس الرئيسي لرصد التقدم المحرز نحو تحقيق هدف التنمية المستدامة 3.4، والذي يقيس نسبة الوفيات المبكرة الناجمة عن الأمراض غير المعدية هو السن (الوفيات بين الأشخاص الذين تتراوح أعمارهم بين 30 و69 عاماً).

**الطريقة:**

لفحص العلاقة بين نسبة الوفيات المبكرة الناجمة عن الأمراض غير المعدية، ونسبة الوفيات الناجمة عن الأمراض غير المعدية لدى الأشخاص الأكبر سناً، تم تجميع قاعدة بيانات لمعدلات الوفيات الناتجة عن أمراض القلب والأوعية الدموية، والسرطان، ومرض الانسداد الرئوي المزمن، والسكري لدى الأشخاص الذين تتراوح أعمارهم بين 30 و69 عاماً، والأشخاص بين 70 و89 عاماً، وذلك باستخدام تقديرات من دراسة العبء العالمي للأمراض سنة 2017. وشملت البيانات 195 دولة، وست نقاط زمنية، وكلا الجنسين، بما يمثل 2340 حالة. تم تطبيق طريقة جدول الحياة، التابع لمنظمة الصحة العالمية (WHO)، لقياس نسبة الوفيات الناجمة عن الأمراض غير المعدية، على البيانات.

**النتائج:**

كان هناك علاقة قوية بين أنماط الوفيات الناجمة عن الأمراض غير المعدية في المجموعتين المبكرة والمتقدمة في السن، مما يشير إلى أن قياس الوفيات المبكرة للأمراض غير السارية يقدم معلومات عن مثل هذه الوفيات في وقت لاحق من الحياة. لم يكن للوقت ولا للموقع الجغرافي تأثير ملموس على هذه العلاقة. ومع ذلك، كان هناك اختلافات بين الإناث والذكور في احتمالات الوفاة المرتبطة بسن محدد، وذلك بسبب الأمراض غير المعدية، مما يعني أن الوفيات الناتجة عن هذا المرض غير المعدي يجب تقييمها باستخدام نهج يعتمد على الجنس.

**الاستنتاج:**

نظراً لأن مقياس منظمة الصحة العالمية المتبع للوفيات المبكرة الناتجة عن الأمراض غير المعدية كان متوقعاً لوفيات الأمراض غير المعدية لدى الأشخاص الأكبر سناً، فإنه لا يجب أن يُفسر المقياس على أنه يعتمد على السن. إن تركيز الموارد على قياس نسبة الوفيات المبكرة الناجمة عن الأمراض غير المعدية، سيكون مناسباً، وبخاصةً في البيئات التي لا يوجد فيها تسجيل مدني شامل للوفيات. يجب ألا يخل هذا النهج بتوفير الخدمات الصحية طوال فترة الحياة.

## 摘要

**目的:**

旨在探讨监测实现可持续发展目标的 3.4 指标，即测量非传染性疾病过早死亡率（30-69 岁人群的死亡率）是否存在年龄歧视。

**方法:**

为了研究非传染性疾病过早死亡率与老年人非传染性疾病死亡率之间的关联性，我们根据《2017 年全球疾病负担研究》中的预测，建立了针对 30-69 岁人群与 70-89 岁人群的心血管疾病、癌症、慢性阻塞性肺病和糖尿病死亡率的数据库。这些数据涵盖了 195 个国家、6 个时间点的男性和女性，提供了 2340 个实例。针对非传染性疾病过早死亡率的世卫组织寿命表法被应用于此类数据。

**结果:**

非传染性疾病过早死亡率和老年人的非传染性疾病死亡率模式之间关联性强，这表明测量非传染性疾病过早死亡率可以提供晚年非传染性疾病死亡率的信息。时间或地理位置不会对此关联性产生实质性的影响。然而，特定年龄段的非传染性疾病死亡率存在男女差异，这意味着应采用区分性别的方法评估非传染性疾病死亡率。

**结论:**

世卫组织制定的非传染性疾病过早死亡率指标是对老年人非传染性疾病死亡率的预测，该指标不应被视作年龄歧视。测量非传染性疾病过早死亡率可适当集中资源，尤其是在暂无民事死亡登记的国家。使用此方法不得有损于全民健康覆盖服务。

## Резюме

**Цель:**

Изучить, является ли дискриминирующим по отношению к пожилым людям подход, связанный с основным измеряемым параметром, определяющим прогресс в достижении цели устойчивого развития 3.4, то есть с измерением показателя преждевременной смертности от неинфекционных заболеваний (смертности среди людей в возрасте 30–69 лет).

**Методы:**

Для изучения взаимосвязи между преждевременной смертностью от неинфекционных заболеваний и смертностью от неинфекционных заболеваний у пожилых людей была составлена база данных по показателям смертности от сердечно-сосудистых заболеваний, рака, хронической обструктивной болезни легких и диабета у людей в возрасте от 30 до 69 лет и у людей в возрасте от 70 до 89 лет. В основу базы данных легли оценки, сделанные в ходе исследования глобального бремени болезней в 2017 году. Данные охватывали 195 стран, шесть временных точек и представителей обоих полов; всего 2340 случаев. Для получения измеряемых показателей преждевременной смертности от неинфекционных заболеваний к данным применялся метод таблиц вероятности дожития, принятый Всемирной организацией здравоохранения (ВОЗ).

**Результаты:**

Была обнаружена тесная взаимосвязь между показателями смертности от неинфекционных заболеваний в группе преждевременной смертности и в группе пожилого возраста, что позволяет предположить информативность измерения преждевременной смертности от неинфекционных заболеваний как индикатора такой смертности в пожилом возрасте. Ни время, ни географическое положение не оказывало существенного влияния на эту взаимосвязь. Однако были обнаружены различия в показателях между мужчинами и женщинами в части вероятности смерти от неинфекционных заболеваний для конкретного возраста, что позволяет предположить необходимость раздельной оценки показателей смертности от неинфекционных заболеваний по половому признаку.

**Выводы:**

Поскольку установленные ВОЗ измеряемые показатели преждевременной смертности от неинфекционных заболеваний позволяют прогнозировать смертность от этих заболеваний в пожилом возрасте, их не следует рассматривать как возрастную дискриминацию пожилых людей. Выделение ресурсов на измерение масштабов преждевременной смертности от неинфекционных заболеваний будет обосновано, в частности, в условиях, где отсутствует всеобщая система регистрации смертей. Такой подход не должен препятствовать предоставлению услуг здравоохранения на всем протяжении жизни человека.

## Introduction

In recent years, increased attention has been given to the concept of premature mortality, particularly that arising from noncommunicable disease.^1^ In 2012, the sixty-fifth World Health Assembly of the World Health Organization (WHO) adopted a global target for the reduction of premature mortality (that is death among people aged 30–69 years) due to noncommunicable disease and subsequently set out life-table methods for measuring and reporting progress towards achieving this target.^2^ Subsequently, further analyses of premature noncommunicable disease mortality paved the way for the adoption of target 3.4 of the United Nations’ sustainable development goals (SDG):^3^ to “reduce by one third premature mortality from noncommunicable diseases through prevention and treatment” by 2030.^4^

Without doubt, morbidity and mortality from noncommunicable disease engender considerable concern worldwide and probably present the greatest challenge to providing health services for adults of all ages in coming decades.^5^ Despite the international consensus on reducing noncommunicable disease mortality, there has been relatively little discussion or analysis of the implications of choosing premature noncommunicable disease mortality as a key metric. Although there is strong evidence that the premature noncommunicable disease mortality reduction target is viable,^3^ much less is known about strategic targets for noncommunicable disease mortality in older people. Indeed, as the highest rates of death due to noncommunicable diseases occur in later life, it might be considered counterintuitive to focus attention on premature noncommunicable disease deaths. Furthermore, given the high burden of noncommunicable disease in later life worldwide (which partially reflects exposure to risk factors in earlier life), focusing on premature mortality may give an impression of institutional ageism within the health sector.^6^ In fact, the choice of premature noncommunicable disease mortality as the metric for monitoring global progress on noncommunicable disease control has been misconstrued as not caring about the health of older people.^7^ Some of this debate may, however, have blurred the important distinction between what makes a good metric and how effective health-care policy should be implemented throughout the life-course.

Global noncommunicable disease mortality rates are a composite of complex variables. For example, in places where health care is suboptimal, poor diagnosis and treatment in younger adults may lead to premature deaths and inadequate public health measures may fail to reduce exposure to important risk factors, thereby leading to death at any age. In contrast, where health care is more comprehensive, the appropriate diagnosis and treatment of chronic noncommunicable diseases will probably postpone some potentially premature noncommunicable disease deaths till later in life. However, regardless of how well their health-care needs have been met and managed, most older adults will eventually die of noncommunicable diseases in any setting.

Rather than merely add to conceptual discussions about the appropriateness of the premature noncommunicable disease mortality metric as the chosen means of tracking progress on noncommunicable diseases in SDG target 3.4, it seems more helpful to evaluate the evidence supporting this metric’s usefulness and scope. Currently, good primary data on cause-specific mortality in older people are scarce for much of the world’s population and global estimates must be based on modelling. Despite their limitations, cause-specific mortality data, by age and sex, are available over time for every country in the world. Using these data, one can objectively consider the potential value of premature noncommunicable disease mortality metrics for understanding noncommunicable disease mortality in older people (that is, older than 70 years). In particular, the following questions can be addressed: (i) How are premature and older noncommunicable disease mortality rates interrelated within populations? (ii) Do relationships between premature and older noncommunicable disease mortality rates differ by sex? and (iii) Are relationships between premature and older noncommunicable disease mortality rates stable over time and geographical location?

The aim of this article was to provide an evidence-based understanding of the strengths and weaknesses of WHO’s premature noncommunicable disease mortality metric for policy and practice. The article does not set out to elucidate patterns or trends in noncommunicable disease mortality per se. The core question is whether measuring premature noncommunicable disease mortality, as mandated by WHO and SDG target 3.4, also provides reasonably useful information on noncommunicable disease mortality in the older population.

## Methods

A database was compiled of mortality rates for cardiovascular disease, cancer, chronic obstructive pulmonary disease and diabetes (the four major noncommunicable diseases identified by WHO for assessing premature mortality) using estimates from the Global Burden of Disease Study 2017 as source material.^2^^,^^8^ This database reflected data modelled from a wide range of sources for all countries over time in a manner that complies with the Guidelines for Accurate and Transparent Health Estimates Reporting.^9^^,^^10^

Data on deaths and mortality was downloaded that covered 195 countries and was divided into: (i) two sex groups; (ii) twelve 5-year age groups from 30 to 89 years; (iii) six 5-year time points within the period covered by Global Burden of Disease estimates (i.e. 1992, 1997, 2002, 2007, 2012 and 2017); and (iv) four disease groups: cardiovascular disease (Global Burden of Disease cause code 410), cancer (code 587), chronic obstructive pulmonary disease (code 491) and diabetes mellitus (code 509). Overall, the total number of data points was 112 320 (i.e. 195 × 2 × 12 × 6 × 4). In addition, data was derived for individual WHO regions by merging data for countries in each region and each country’s population was calculated as the number of reported deaths × 1000 divided by the country’s mortality rate. The data set used is available in a GitHub data repository (GitHub, San Francisco, United States of America).^11^

These data were used to produce a descriptive summary of the number of deaths and mortality, by year, sex, WHO region and noncommunicable disease cause of death, for two age groups in which deaths due to a noncommunicable disease occur: a premature age group which included people aged 30–69 years and an older age group which included those aged 70–89 years (Table 1). Data for these two age groups were derived by combining data from the relevant 5-year age groups for the categories: (i) year; (ii) sex; (iii) WHO region; and (iv) noncommunicable disease cause of death.

**Table 1:** Deaths and mortality due to four major noncommunicable diseases,^a^ worldwide, 2017

Category	No. of deaths, millions (%)^b^	Mortality, per 1000 people
**Premature age group (30–69 years)**		
Total	63.33 (100)	3.93
Year		
1992	9.40 (14.8)	4.62
1997	9.90 (15.6)	4.34
2002	10.13 (16.0)	3.99
2007	10.27 (16.2)	3.66
2012	11.08 (17.5)	3.61
2017	12.55 (19.8)	3.72
Sex		
Female	25.11 (39.7)	3.12
Male	38.21 (60.3)	4.74
WHO region		
African	4.19 (6.6)	3.29
Americas	7.40 (11.7)	3.28
South-East Asia	15.67 (24.7)	4.16
European	13.12 (20.7)	4.92
Eastern Mediterranean	4.36 (7.0)	4.26
Western Pacific	18.59 (29.4)	3.63
Cause of death		
Cardiovascular disease	32.10 (50.7)	1.99
Cancer	23.58 (37.2)	1.46
Chronic obstructive pulmonary disease	4.86 (7.7)	0.30
Diabetes	2.79 (4.4)	0.17
**Older age group (70–89 years)**		
Total	79.36 (100)	43.55
Year		
1992	10.62 (13.4)	51.15
1997	11.52 (14.5)	48.11
2002	12.72 (16.0)	45.71
2007	13.47 (17.0)	42.04
2012	14.88 (18.8)	40.80
2017	16.15 (20.4)	39.22
Sex		
Female	39.39 (49.6)	38.09
Male	39.97 (50.4)	50.72
WHO region		
African	3.55 (4.3)	40.73
Americas	10.74 (13.5)	35.36
South-East Asia	13.07 (16.5)	43.58
European	22.44 (28.4)	46.05
Eastern Mediterranean	3.52 (4.6)	51.46
Western Pacific	26.04 (32.6)	45.22
Cause of death		
Cardiovascular disease	47.02 (59.2)	25.81
Cancer	18.59 (23.4)	10.20
Chronic obstructive pulmonary disease	11.01 (13.9)	6.04
Diabetes	2.73 (3.4)	1.50

WHO: World Health Organization.

^a^ The four noncommunicable diseases identified by the World Health Organization for assessing premature mortality were cardiovascular disease, cancer, chronic obstructive pulmonary disease and diabetes mellitus.

^b^ Percentages are of deaths in each age group.

Data source: The Global Burden of Disease Study 2017.^8^

Subsequently, WHO’s life-table method for the premature noncommunicable disease mortality metric was applied to the whole data set.^2^ The noncommunicable disease mortality rate at each 5-year time-point for each age group (i.e. *x* to *x*+5 years), *_5_M_x_*, was estimated for each country and year of observation by summing relevant mortality rates for the four noncommunicable disease causes of death. These estimates of *_5_M_x_* were then used to calculate corresponding probabilities of death due to a noncommunicable disease over 5-year intervals, *_5_q_x_*:
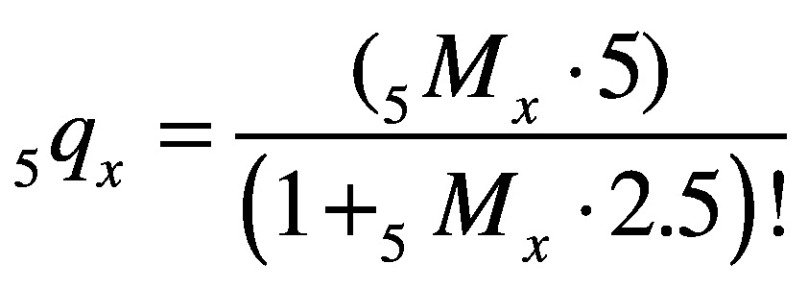
(1)Unconditional probabilities of death due to a noncommunicable disease for the 30 to 69-year age group (*_40_q_30_*) and the 70 to 89-year age group (*_20_q_70_*), for each country, sex and 5-year time-point, were then calculated using these 5-year probabilities of death, as: 
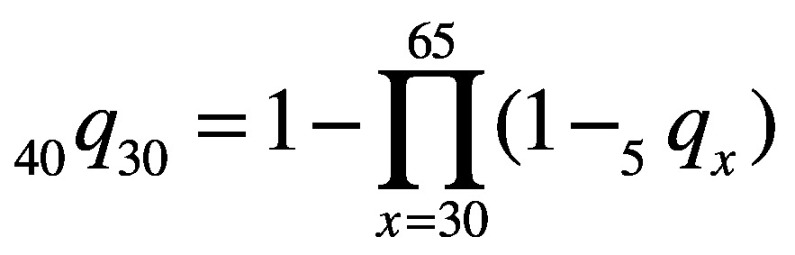
(2)and 


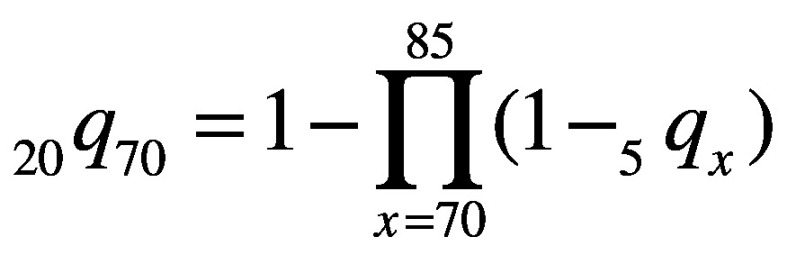
(3)

The magnitude of the probability of death due to a noncommunicable disease in the older age group relative to the premature age group, for each country, sex and time-point, was defined as the ratio of *_20_q_70_* to *_40_q_30_*. The resulting data set contained 2340 unique instances each of *_40_q_30_*, *_20_q_70_* and *_20_q_70_* : *_40_q_30_*, which were subsequently analysed by WHO region, country, sex and year.

The Stata curvefit command (StataCorp LP., College Station, USA) was used to examine the relationship between the probability of death due to a noncommunicable disease in the premature age group (*_40_q_30_*) and the ratio of the probability of death between the older and premature age groups (*_20_q_70_* : *_40_q_30_*) by WHO region, sex and year. The curve with the best fit was identified to be a power function. All calculations were performed in Stata v. 12.1.

## Results

 In 2017, mortality rates in the 70 to 89-year age group were an order of magnitude higher than those in the 30 to 69-year age group (Table 1). Although the numbers of deaths in the two groups were comparable, there were fewer people in the older age group as this group made up a much lower proportion of the population. In general, mortality rates decreased modestly over time, were higher among males and were highest in WHO European and Eastern Mediterranean Regions. They were predominantly attributable to cardiovascular disease and cancer, out of the four noncommunicable diseases studied.

On applying WHO’s method for the premature noncommunicable disease mortality metric to the data,^2^ The probability of death due to a noncommunicable disease in the premature age group varied by sex, country and year from 0.050 for females in the Republic of Korea in 2017 to 0.533 for males in Papua New Guinea in 1992; the median was 0.207. The probability of death in the older age group varied from 0.274 for females in Singapore in 2017 to 0.983 for males in Uzbekistan in 2012; the median was 0.695. The ratio of the probabilities of death due to a noncommunicable disease between the older and premature age group, ranged from 1.57 for males in Papua New Guinea in 1992 to 7.67 for females in Greece in 2002; the median was 3.28.

**Fig. 1:**
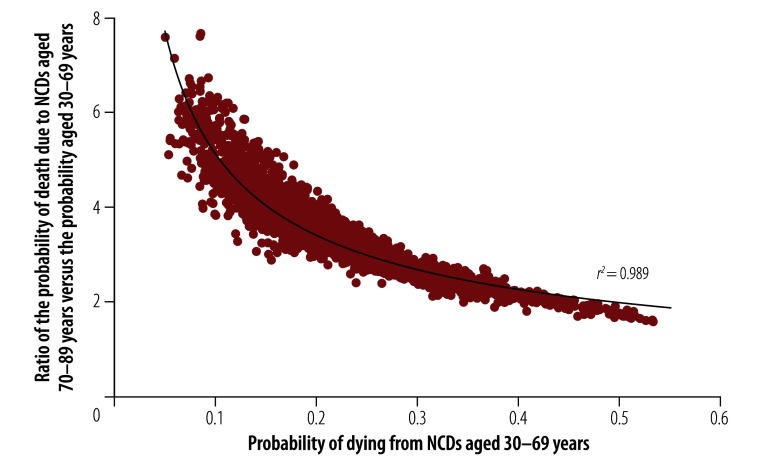
The probability of noncommunicable disease deaths in those aged 30 to 69 years and the ratio of the probability of these deaths in those aged 70 to 89 years compared to 30 to 69 years, worldwide, 2017

Notes: The four noncommunicable diseases identified by the World Health Organization for assessing premature mortality were cardiovascular disease, cancer, chronic obstructive pulmonary disease and diabetes mellitus. The curve represents the line of best fit.

The correlation between probabilities of death in the premature age group and the older : premature ratio over all 2340 country, year and sex combinations is shown in Fig. 1 and has an *r^2^* value of 0.989. The fitted curve can be expressed by the equation: 

Data source: The Global Burden of Disease Study 2017.^8^


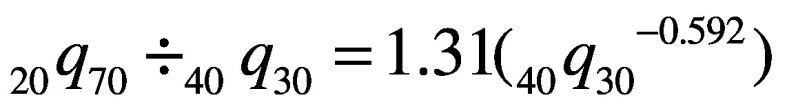
(4)

The shape of the curve in Fig. 1 shows that, in addition to the strong correlation between the probabilities of death in the premature age group and the older: premature probability ratio, lower values in the premature age group correspond to proportionately higher values of the ratio.

**Fig. 2:**
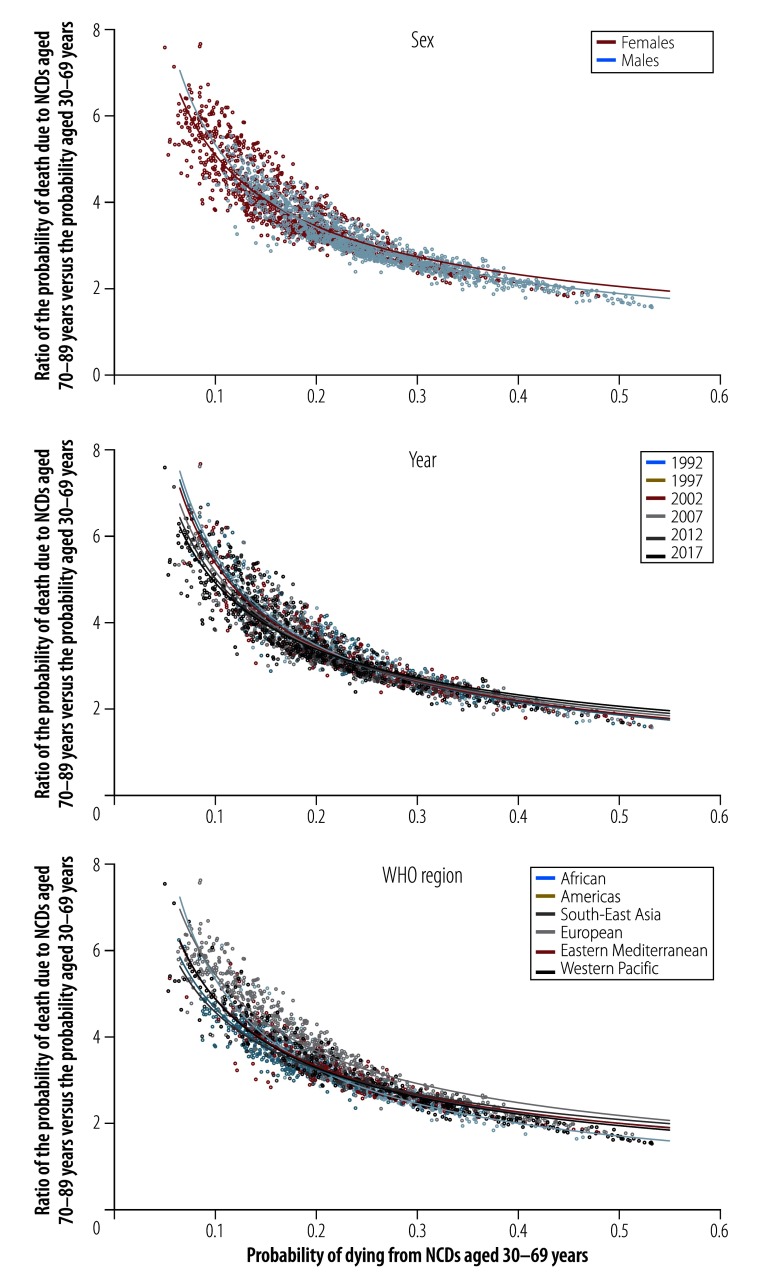
The probability of noncommunicable disease deaths in those aged 30 to 69 years and the ratio of the probability of these deaths in those aged 70 to 89 years compared to 30 to 69 years, by sex, year and WHO region

Fig. 2 shows similar scatter plots of probabilities of death for the 2340 data points categorized by sex, year and WHO region, respectively, and includes separate fitted curves for each subgroup. The median probability of death for the premature age group for females over all countries and years was 0.178; the corresponding value for males was 0.243. The median older : premature ratio was 3.65 for females and 2.98 for males. Analysed by year, the median value for the premature age group decreased from 0.229 in 1992 to 0.180 in 2017, whereas the median older : premature ratio increased from 3.11 in 1992 to 3.53 in 2017. Analysed by WHO region, the median probability for the premature age group ranged from 0.173 for the Region of the Americas to 0.251 for the Western Pacific Region and the median older : premature ratio ranged from 2.97 for the Western Pacific Region to 4.03 for the European Region.

WHO: World Health Organization.

Notes: The four noncommunicable diseases identified by WHO for assessing premature mortality were cardiovascular disease, cancer, chronic obstructive pulmonary disease and diabetes mellitus. The curves show the lines of best fit for each subgroup.

Data source: The Global Burden of Disease Study 2017.^8^

## Discussion

Globally, a strong relationship was found between the probability of death due to four major noncommunicable diseases in the 70 to 89-year age group and the probability in the 30 to 69-year age group. This observation has important implications for noncommunicable disease policy and practice, since premature noncommunicable disease mortality is a key metric for WHO noncommunicable disease reduction strategies and SDG target 3.4, as health care throughout the life-course is a key deliverable. Although the absolute numbers of deaths due to a noncommunicable disease in the premature and older age groups were very similar globally, the biological reality is that the probability of dying in general, and from a noncommunicable disease in particular, is strongly age-related. Estimates from the Global Burden of Disease study indicate that there has been some success in reducing the noncommunicable disease mortality rate in the older age group over time: from 51.15 to 39.22 per 1000 globally between 1992 and 2017. Nevertheless, rates in the older group remained an order of magnitude higher than those in the premature age group, which fell similarly from 4.62 to 3.72 per 1000 over the same period.

Population ageing, which is occurring in many societies, is being driven by the successful postponement of death, particularly death due to a noncommunicable disease. Nevertheless, the median probability of death observed for the older age group reflects the fact that most people who reach 70 years of age die of a noncommunicable disease before their 90th birthday. Thereafter, only a small proportion of survivors 90 years of age go on to celebrate their centenaries. The correlation between probability of death in the premature group and the older: premature ratio suggests that, although there may be scope for reducing the probability of death from a noncommunicable disease in general, reducing the probability of dying from a noncommunicable disease at an older age is likely to be less tractable, even if premature noncommunicable disease mortality is substantially reduced. Consequently, as the proportion of people in the world who survive till their seventieth birthday increases, in future there is likely to be a higher number of deaths due to a noncommunicable disease in the older age group. This should not, therefore, be regarded as a public health failure in addressing noncommunicable diseases.

With any population health target, considering how various subgroups might be affected is required. In this study, the most obvious variation was by sex, with females having generally lower probabilities of death from a noncommunicable disease. This result is consistent with adult females’ overall survival advantage, which is well established almost everywhere in the world, particularly in older age groups. Variations by year and WHO region were less pronounced and these variables do not seem to be major cofactors influencing the relationship between probabilities of death in the premature and the older age groups. Such relationships suggest that the premature noncommunicable disease mortality metric provides very useful, indirect, but generalizable information about noncommunicable disease mortality in older adults.

In practice, monitoring cause-specific mortality among older people successfully can be difficult, especially in areas where death certification is not mandatory. In particular, the cause of death in older people is often uncertain when verbal autopsies are used. For example, in an analysis of over 100 000 deaths across Africa and Asia,^12^ the cause of death remained undetermined in 14.6% (4 364/29 911) of people older than 65 years, compared with 11.5% (7 862/68 518) of those younger than 65 years. Clinically, the cause of death may be less clear in older people with multiple morbidities and culturally, verbal autopsy informants may be less forthcoming about the circumstances of the death if there is an underlying belief that “she died because she was old.” Therefore, concentrating efforts on improved monitoring of the cause of death in people younger than 70 years of age may be more realistic in settings with constrained resources.

This study has limitations. The need to base the analysis on modelled global estimates was not ideal. Although Global Burden of Disease estimates may be good, underlying source data, particularly from low- and middle-income countries, remain scarce.^13^ In addition, the Global Burden of Disease modelling process is so complex one cannot be sure that undertaking further analyses based on those modelled outputs does not somehow reveal underlying assumptions as artefacts. However, given its global scope, the analysis included a considerable amount of data from countries with excellent information systems that were much less likely to be affected by the modelling process. Consequently, the risk of uncovering modelling artefacts was reduced.

In conclusion, the close relationship between noncommunicable disease mortality rates in people younger and older than 70 years of age shown in this study, suggests that using premature noncommunicable disease mortality as a metric is not in itself an ageist strategy. Use of the metric, as required by SDG target 3.4, neither involves nor implies any lack of care or concern for older people and their health; to the contrary, premature noncommunicable disease mortality rates can, as shown here, predict rates among older people. Moreover, in terms of care of older people, declarations by WHO and other agencies have frequently stated their commitment to universal health coverage, which by definition covers the whole life-course.^14^

The unsurprising female-to-male differences observed make it essential that assessments of noncommunicable disease mortality should adopt a sex-disaggregated approach, not least to avoid the allegation that noncommunicable disease mortality targets are sexist. In a world of scarce resources, the accurate assessment of premature noncommunicable disease mortality is a pragmatic strategy for tracking overall progress on tackling noncommunicable diseases. The major remaining practical challenge is to strengthen on-the-ground information systems so that premature noncommunicable disease mortality can be monitored directly and reliance on modelled estimates, as used here, can be reduced.
